# Ligand-Free Silver Nanoparticles: An Innovative Strategy against Viruses and Bacteria

**DOI:** 10.3390/microorganisms12040820

**Published:** 2024-04-18

**Authors:** Maria Vittoria Morone, Annalisa Chianese, Federica Dell’Annunziata, Veronica Folliero, Erwin Pavel Lamparelli, Giovanna Della Porta, Carla Zannella, Anna De Filippis, Gianluigi Franci, Massimiliano Galdiero, Antonio Morone

**Affiliations:** 1 Department of Experimental Medicine, Section of Microbiology and Clinical Microbiology, University of Campania “L. Vanvitelli”, 80138 Naples, Italy; mariavittoria.morone@unicampania.it (M.V.M.); annalisa.chianese@unicampania.it (A.C.); federica.dellannunziata@unicampania.it (F.D.); carla.zannella@unicampania.it (C.Z.); anna.defilippis@unicampania.it (A.D.F.); massimiliano.galdiero@unicampania.it (M.G.); 2 Department of Medicine, Surgery and Dentistry “Scuola Medica Salernitana”, 84081 Baronissi, Italy; vfolliero@unisa.it (V.F.); elamparelli@unisa.it (E.P.L.); gdellaporta@unisa.it (G.D.P.); gfranci@unisa.it (G.F.); 3 Interdepartment Centre BIONAM, Università di Salerno, via Giovanni Paolo I, 84084 Fisciano, Italy; 4 Consiglio Nazionale delle Ricerche, Instituto di Struttura della Materia U.O. di Tito Scalo, 85050 Potenza, Italy

**Keywords:** PLAL-AgNPs, antiviral activity, antibacterial activity, antibiofilm activity, silver nanoparticles, multidrug resistance, poliovirus, herpesvirus

## Abstract

The spread of antibiotic-resistant bacteria and the rise of emerging and re-emerging viruses in recent years constitute significant public health problems. Therefore, it is necessary to develop new antimicrobial strategies to overcome these challenges. Herein, we describe an innovative method to synthesize ligand-free silver nanoparticles by Pulsed Laser Ablation in Liquid (PLAL-AgNPs). Thus produced, nanoparticles were characterized by total X-ray fluorescence, zeta potential analysis, transmission electron microscopy (TEM), and nanoparticle tracking analysis (NTA). A 3-(4,5-dimethylthiazol-2-yl)-2,5-diphenyltetrazolium bromide (MTT) assay was performed to evaluate the nanoparticles’ cytotoxicity. Their potential was evaluated against the enveloped herpes simplex virus type 1 (HSV-1) and the naked poliovirus type 1 (PV-1) by plaque reduction assays and confirmed by real-time PCR and fluorescence microscopy, showing that nanoparticles interfered with the early stage of infection. Their action was also examined against different bacteria. We observed that the PLAL-AgNPs exerted a strong effect against both methicillin-resistant *Staphylococcus aureus* (*S. aureus* MRSA) and *Escherichia coli* (*E. coli*) producing extended-spectrum β-lactamase (ESBL). In detail, the PLAL-AgNPs exhibited a bacteriostatic action against *S. aureus* and a bactericidal activity against *E. coli*. Finally, we proved that the PLAL-AgNPs were able to inhibit/degrade the biofilm of *S. aureus* and *E. coli*.

## 1. Introduction

Infectious diseases encompass a variety of disorders caused by a broad range of pathogens, such as viruses, bacteria, fungi, and parasites, that affect human health [1]. These types of infections are some of the most significant causes of illness, disability, and death in the world.

According to the World Health Organization (WHO), infectious diseases account for 90% of health problems and kill about 14 million people every year, 90% of whom come from developing countries [2]. The high ability of microorganisms to mutate over time causes the onset of resistance to antimicrobial drugs, and therefore new therapeutic strategies are necessary to improve not only the rate of healing but also the quality of life of patients [3]. Drug-resistance phenomena are frequent in cases of herpes simplex virus type 1 (HSV-1) infections [4]. These diseases are effectively treated with acyclovir, which is the most-used drug. However, drug-resistant strains are recurrent, mainly among bone marrow transplant recipients and immunocompromised people, with a prevalence of about 30% and 5%, respectively [5]. As well as causing recurrent oral–facial lesions, HSV-1 infections are the leading cause of viral encephalitis [6]. It is estimated that 60–80% of the world population is infected by HSV-1, depending on age and socio-economic status [7]. Given the high prevalence of the infection, clinical complications, and the development of drug resistance, new and effective antiviral drugs are needed. 

In addition to infections caused by HSV-1, poliovirus type 1 (PV-1), an enterovirus belonging to the *Picornaviridae* family [8], causes several human and animal diseases ranging from severe (poliomyelitis, encephalitis, meningitis, and apatite) to mild (common cold) affections [9]. There is no cure for poliomyelitis, and its prevention relies solely on immunization. Currently, two types of vaccines are available: oral and inactivated polio vaccines. Both types of vaccines are widely recognized for their effectiveness and safety, and they are used in various combinations worldwide to ensure optimal protection for the population. However, the endemic transmission of poliovirus continues in some areas of the world, such as Afghanistan and Pakistan [10]. The inability to stop this type of infection could lead to the global resurgence of the disease. 

On the other hand, documented in almost every part of the world are the infections due to multidrug-resistant (MDR) bacteria, which are widely considered to represent a silent global pandemic [11]. For instance, *Escherichia coli* (*E. coli ESBL*) resistant to third-generation cephalosporins and methicillin-resistant *Staphylococcus aureus* (*S. aureus* MRSA) are included in critical and medium risk categories [12]. The use of non-traditional antibacterial drugs could be a promising solution to prevent the development of resistant bacteria as against common antibiotics [13,14]. 

Nanotechnology represents a rapidly growing science that has great potential in various applicative fields, such as drug development, tissue engineering, decontamination processes, information technologies, and manufacturing light and/or resistant materials [15]. Several studies have reported that nanoparticles can be used as valid and new tools to control the phenomenon of drug resistance [16,17,18]. Among the nanoparticles of great interest are silver nanoparticles (AgNPs), extensively described for their excellent antimicrobial properties [19]. AgNPs are nanomaterials consisting of 20–15,000 silver atoms, with a diameter of 1–100 nm [20], a high surface-to-volume ratio, and a free surface area [21,22,23]. Various methods, including chemical, physical–biological, and hybrid techniques, can be adapted to manufacture nano- and microsilver particles by either physical or chemical approaches. Physical methods of synthesis include plasma arcing, ball milling Pulsed Laser Ablation in Liquid (PLAL), spray pyrolysis, and lithographic techniques, while chemical approaches include the electro-deposition method, the sol–gel process chemical solution method, chemical vapor deposition methods, hydrolysis co-precipitation, and the wet chemical method [24]. Chemical synthesis has the advantage of high yields of nanoparticles, but the disadvantage lies in controlling the morphology of the nanoparticles formed. On the contrary, physical approaches yield a lower number of nanoparticles but allow for excellent control of their morphology [25].

In detail, PLAL is a top-down physical approach that allows the production of ligand-free nanoparticles of noble metals with different optical and morphological properties [26].

This methodology, due to the control of laser energy, allows the production of nanoparticles with spherical symmetry; in addition, the size of AgNPs (10–100 nm) and their non-aggregated status ensure relevant antimicrobial activity [27]. The high fluence used in this approach prevents oxidation of the silver target and therefore ensures the metallicity of the resultant AgNPs [28].

In the present study, AgNPs were synthesized by PLAL; for simplicity, they are called PLAL-AgNPs throughout the text. They were characterized by total X-ray fluorescence, zeta potential, transmission electron microscopy (TEM), and nanoparticle tracking analysis (NTA); finally, they were analyzed for their antiviral, antibacterial, and antibiofilm potential.

## 2. Materials and Methods

### 2.1. Methods for Production of AgNPs

The Pulsed Laser Ablation in Liquid (PLAL) technique was used to produce Ag nanoparticles starting from a chemically pure Ag target (99.99%). In our laboratory, we operated with a Neodymium-Yag-Laser (YAG) with the following features: a wavelength of 532 nm, a duration pulse time of 10 ns, and a frequency repetition rate of 10 Hz.

The laser beam was focused on an area of 3 mm^2^ of the Ag target, and the fluence value was 0.8 J/cm^2^. As regards the geometric set-up, the laser beam impinged on the target surface, forming an angle of 90° with respect to the surface of the Ag target. 

The target material was inserted in a quartz cuvette, and it was filled with 10 mL phosphate-buffered saline (PBS) 1×. During the PLAL process, an atmospheric pressure equal to 760 mm Hg, a temperature equal to 23 °C, and a pulse duration equal to 120 min were used [29].

**Figure 1:**
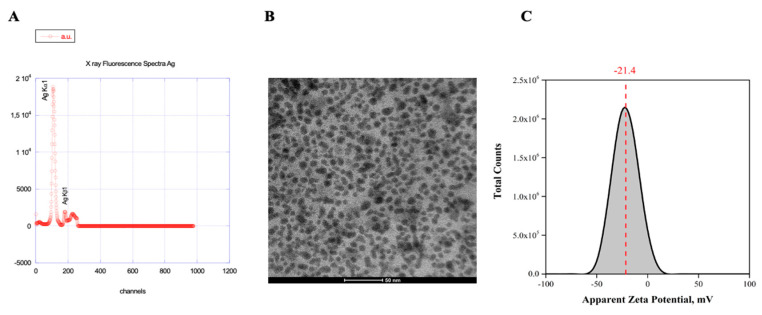
Physical–chemical characterization. (**A**) X-ray fluorescence spectrum of Ag. The characteristic X-ray emission lines of the Ag target used to produce PLAL-AgNPs are present, while no other material is visible, demonstrating the purity of the Ag target. (**B**) TEM visualization of PLAL-AgNPs at 50 nm magnification. (**C**) Zeta potential analysis of PLAL-AgNPs. The zeta potential value of the PLAL-AgNPs is −21.4 mV.

In order to evaluate the purity of the Ag target material used during the Pulsed Laser Ablation in Liquid procedure, X-ray fluorescence spectra were determined. In the spectrum in Figure 1, only X-ray emission lines of Ag are characteristics of our target. This analysis was very important because it excluded the presence of other materials in the Ag target and guaranteed its purity.

### 2.2. PLAL-AgNP Characterization

#### 2.2.1. Total X-ray Fluorescence

We used an Amptek system to collect the total X-ray fluorescence spectra. It was composed of an X-ray source that worked with an Ag anode, and the fluorescence X-ray lines were obtained using a Si-PIN photodiode detector; the energy resolution was 149 eV, and the detection area was 13 mm^2^. The collected signals were processed through a multichannel analyzer, and the data were obtained using the Amptek software version 3.0.

#### 2.2.2. Transmission Electron Microscopy (TEM) Analysis

The morphology and size of the AgNPs were confirmed by TEM analysis. In particular, the PLAL-AgNPs were deposited on a carbon-coated copper grid on which a 2% phosphotungstic acid solution was added. The excess solution was gently aspirated using filter paper. The grid thus prepared was left to dry overnight before TEM analysis at a 200 kV voltage. The procedure was carried out using an FEI tecnai G2, and images were captured using an FEI TEM (version 4.7 SP3).

#### 2.2.3. Zeta Potential

To evaluate the stability of the PLAL-AgNP solution, the ζ-potential was measured using the electro-phoretic light scattering method (ELS) by a Zetasizer Nano S instrument (Malvern PANalytical, Malvern, UK). ζ-potential measurements were performed at room temperature using disposable cuvettes (DTS1070, Malvern PANalytical) filled with 1 mL of the sample. All measurements were repeated three times, with a 60 s equilibration step between each measurement. The software version 7.10 was set to the automatic acquisition mode.

#### 2.2.4. UV-Vis

A spectrophotometer (Varian Cary 50 UV-Vis) was used to measure the optical absorption spectra and assess the absorption of electromagnetic radiation across the ultraviolet and visible ranges. Specifically, the spectra of silver nanoparticles were recorded using a quartz cuvette, scanning continuously from 200 to 800 nm. Phosphate-buffered saline (PBS) served as the reference for baseline correction.

#### 2.2.5. Determination of AgNP Concentration Nanoparticle Tracking Analysis (NTA)

The concentration of PLAL-AgNPs was determined using a NanoSight NS300 nanoparticle tracking instrument (NTA) (Malvern Panalytical, Worcestershire, UK). A sample of PLAL-AgNPs for measurement was diluted 1:4 in PBS and inserted into the instrument through a pump syringe at a speed of “125” and a level chamber of “12”. Five readings of 60 s each were taken at room temperature (18.0–18.2 °C). A total of 1498 frames per sample were analyzed by NTA software (Malvern Instruments, version 3.2, Worcestershire, UK) with a detection threshold of 4.

### 2.3. Cell Lines and Viral Strains

Vero-76 cells (American Type Culture Collection, ATCC CRL-1587, Manassas, VA, USA) were cultured in Dulbecco’s Modified Eagle Medium (DMEM) (Microtech, Naples, Italy) with 4.5 g/L glucose, 2 mM L-glutamine, and 100 IU/mL penicillin/streptomycin solution (Himedia, Naples, Italy) supplemented with 10% fetal bovine serum (FBS) (Microtech, Naples, Italy) in a humidified atmosphere with 5% CO_2_ at 37 °C. HSV-1 (strain SC16), HSV-1 GFP (containing the GFP reporter inserted into the gene coding for the VP22 tegument protein [30]), and Sb-1 (poliovirus Sabin strain Chat ATCC VR-1562) were grown on Vero cells as previously described [31].

### 2.4. Bacteria and Growth Conditions

The bacterial strains used in this study were purchased from the ATCC. *E. coli (*ATCC 11229 non-biofilm producer, ATCC 25922 biofilm producer) and *S. aureus* (ATCC 6538 non-biofilm producer, ATCC 25923 biofilm producer) were chosen as representatives of Gram-negative and -positive bacteria, respectively. Furthermore, two clinical isolates (*E. coli* Extended Spectrum Beta-Lactamase (ESBL) and multidrug-resistant (MDR) *S. aureus*) were collected at the University Hospital of Campania “Luigi Vanvitelli” (Naples, Italy). To evaluate the antimicrobial effect of PLAL-AgNPs, bacteria were seeded on Mueller–Hinton (MH) agar plates (Oxoid, Hampshire, MA, USA) at 37 °C for 24 h. Then, the tests were performed by incubating a single colony overnight (ON) in MH broth (Sigma-Aldrich, St. Louis, MO, USA) at 37 °C under orbital shaking (180 rpm). To evaluate the AgNPs’ degradative/inhibitory efficacy on biofilm, the strains were seeded on Luria–Bertani (LB) agar plates (Oxoid, Hampshire, MA, USA) and inoculated in LB broth (Sigma-Aldrich, St. Louis, MO, USA).

### 2.5. Cell Viability

The cytotoxic effect of the AgNPs was evaluated by the 3-(4,5-dimethylthiazol-2-yl)-2,5-diphenyltetrazolium bromide (MTT) test. Cells were seeded into 96-well plates at a density of 2 × 10^4^ cells/well and incubated at 37 °C with 5% CO_2_ in a humid environment for 24 h. Then, the cell monolayer was exposed to 2.3 × 10^7^ to 1.8 × 10^5^ PLAL-AgNPs at 37 °C for 24 h. As a negative control (ctr-), 100% DMSO was used, while culture medium was used as the positive control (ctr+). After exposure, the medium was removed and 0.5 mg/mL of MTT solution (Sigma-Aldrich, St. Louis, MO, USA) was added to each well for 3 h. Formazan crystals were solubilized by adding 100% DMSO, and cell viability was determined by measuring the absorbance at 570 nm through a TECAN M-200 reader (Tecan, Männedorf, Switzerland). The percentage of cell viability was calculated according to the following formula:

$$Cell\;viability(\%)=\frac{absorbance\;of\;treated\;samples}{absorbance\;of\;not\;treated\;cell}\;\times 100$$

### 2.6. Antiviral Activity

To evaluate the antiviral properties of the PLAL-AgNPs, different plaque assays were performed against selected viruses, including HSV-1, GFP HSV-1, and PV-1. 

Cells were seeded in 12-well plates at a density of 2 × 10^5^ cells/well and incubated at 37 °C with 5% CO_2_ for 24 h. 

In the co-treatment assay, cell monolayers were simultaneously exposed to PLAL-AgNPs in quantities ranging from 1.2 × 10^7^ to 1.6 × 10^6^ and infected with the virus at a multiplicity of infection (MOI) of 0.01 for 1 h at 37 °C.

In the virus pre-treatment assay, PLAL-AgNPs were directly exposed to a viral suspension at an MOI of 0.1 for 1 h at 37 °C. Subsequently, the mixture was diluted on cells for another hour at 37 °C.

In the cell pre-treatment assay, AgNPs were exposed to cell monolayers for 1 h, and, after that, the cells were infected with the virus at an MOI of 0.01 for another hour at 37 °C.

In the post-treatment assay, cells were first infected with the viral suspension at an MOI of 0.01 per 1 h at 37° C and afterward they were treated with PLAL-AgNPs for 1 h at 37 °C. 

At the end of each treatment, the cell monolayer was washed with citrate buffer (pH 3) (Sigma-Aldrich, St. Louis, MO, USA) and incubated with DMEM supplemented with carboxymethylcellulose (CMC) 3% (Sigma-Aldrich, St. Louis, MO, USA) for 48 h. Subsequently, the cell monolayer was fixed with 4% formaldehyde (Sigma-Aldrich, St. Louis, MO, USA) and stained with 0.5% crystal violet (CV) (Sigma-Aldrich, St. Louis, MO, USA). The viral plaques were counted, and the percentage of inhibition was calculated with respect to infected cells (ctrl-) as follows:

$$Antiviral\;activity(\%)=1-\frac{plaques\;counted\;in\;treated\;cells}{plaques\;counted\;in\;infected\;cells}\;\times 100$$

### 2.7. Molecular Analysis

The antiviral efficacy of PLAL-AgNPs was also investigated by molecular testing in order to confirm the results obtained by plaque reduction assays. The virus pre-treatment assay was performed as described previously. RNA extract was collected by TRIzol (Thermo Fisher, Waltham, MA, USA) and transcribed into cDNA (SensiFAST™ cDNA Synthesis Kit, Meridian Bioscience, Washington, DC, USA). Viral gene expression was evaluated. In detail, for HSV-1, the expression of immediate early (IE, UL54: forward, 5′-TGGCGGACATTAAGGACATTG-3′; reverse, 3′-TGGGCCGTCAACTCGCAG-5′), early (E, UL52: forward, 5′-GACCGACGGGTGCGTTATT-3′; reverse, 3′-GAAGGAGTCGCCATTTAGCC-5′), and late (L, UL27: forward, 5′-GCCTTCTTCGCCTTTCGC-3′; reverse, 3′-CGCTCGTGCCCTTCTTCTT-5′) genes was quantified, while for poliovirus, the expression of the capsid protein VP1 (forward, 5′-GTGCATGCGTGGCCATTATA-3′; reverse, 3′AGACTAACAATGGGCAT-5′) was analyzed. Glyceraldehyde 3-phosphate dehydrogenase (GAPDH, forward, 5′-CCTTTCATTGAGCTCCAT-3′; reverse, 3′-CGTACTGGGAGCGTC-5′) was used as a housekeeping gene to normalize the target threshold cycle (Ct). Gene expression was examined by calculating 2^−ΔΔCt^ values. 

### 2.8. Antibacterial Activity

#### 2.8.1. Microdilution Assay

The PLAL-AgNPs’ antibacterial potential was evaluated through a microdilution assay according to the National Committee on Clinical Laboratory Standards (NCCLS). PLAL-AgNP dilutions (from 1.2 × 10^7^ to 3.6 × 10^5^) were prepared in MH broth. The microdilution assay was performed in a 96-well plate; 50 μL of the standardized bacteria inoculum (5 × 10^5^ CFU/mL) was added to each well. In the assays, vancomycin (2 μg/mL) was used as a positive control for Gram-positive bacteria, while amikacin (8 μg/mL) was used as a positive control for Gram-negative bacteria. The growth rate was assessed after 20 h by the TECAN M-200 reader (Tecan, Männedorf, Switzerland) at OD600, and the minimum inhibitory concentration (MIC) was calculated.

#### 2.8.2. Time–Killing Analysis

Bacterial growth was monitored over time in response to PLAL-AgNP treatment at ½ × MIC and 1 × MIC. Untreated and treated bacteria were used as the ctrl- and the ctrl+, respectively. Inocula of the bacteria were added to each tube and incubated at 37 °C overnight (ON). At regular intervals of 0, 2, 4, 6, and 20 h, 100 μL of bacterial suspension from each sample was serially diluted in PBS 1× and seeded on MH agar plates. After ON incubation at 37 °C, the bacterial colonies were counted.

#### 2.8.3. Biofilm Inhibition and Degradation

The ability of PLAL-AgNPs to inhibit biofilm formation and degradation was evaluated by colorimetric assay. Antibiofilm activity was investigated against Gram-positive (*S. aureus*) and Gram-negative (*E. coli*) bacteria. For both assays, the bacterial inoculum grown overnight was diluted up to 2 × 10^8^ CFU/mL in LB supplemented with 1% glucose. For biofilm inhibition, 100 μL of the bacterial suspension was added to each well simultaneously with AgNPs (1.2 × 10^7^–3.6 × 10^5^). For the mature biofilm degradation, 100 μL of the bacterial suspension was added to each well of the 96-well plate and incubated at 37 °C for 24 h. Subsequently, planktonic cells were removed by washing them twice with PBS 1X, and the mature biofilm was treated with PLAL-AgNPs. As positive and negative controls, vancomycin (20 μg/mL) was used for Gram-positive bacteria and amikacin (80 μg/mL) was used for Gram-negative bacteria, while untreated bacteria represented the negative control. In both assays, after exposure, the biofilm was washed with 1X PBS and stained with 0.1% Cristal violet for 30 min at room temperature (RT). The absorbance was measured at 570 nm by a TECAN instrument, and the percentage of inhibition was analyzed. 

### 2.9. Scanning Electron Microscopy (SEM) Analysis

The morphological changes induced after PLAL-AgNP treatment were estimated by SEM analysis. *E. coli* and *S. aureus* ATCC were treated with the compound at ½ × MIC and 1 × MIC. Untreated and antibiotic-treated bacteria represented the ctrl- and the ctrl+, respectively. Subsequently, the bacteria were fixed in 2.5% glutaraldehyde and dehydrated with ethanol solutions at increasing concentrations (25, 50, 70, 95, and 100% *v*/*v*). Images were acquired using a ZEISS Supra 40 at an accelerating voltage of 5 kV with an Everhart Thornley Detector (ETD) and a Through Lens Detector (TLD) set at 10,000, 20,000, and 50,000× magnifications (EHT = 5.00 kV, WD = 22 mm, the detector in the objective) (Berlin, Germany).

### 2.10. Statistical Analysis

Data were expressed as means ± standard deviations (SDs). The significance of the difference between treated samples and the ctrl- was determined with ANOVA statistics and Dunnett’s test using Graph Pad Software Prism 9.0 (San Diego, CA, USA). A *p*-value < 0.05 was considered significant. All experiments were performed in triplicate. 

## 3. Results and Discussion

### 3.1. PLAL-AgNP Characterization

X-ray fluorescence spectra were determined to evaluate the purity of the Ag target used during the PLAL procedure. In the spectrum in Figure 1A, only Ag X-ray emission lines have the target characteristics. This analysis was crucial to exclude the presence of other materials in the Ag target and to ensure its purity.

The TEM analysis was carried out to appreciate the size and morphology of the synthesized PLAL-AgNPs, as shown in Figure 1B. The NPs appeared spherical, with an average size of 10–100 nm.

Zeta potential analysis was conducted (as described above) to assess the stability of the PLAL-AgNP solution and the nanoparticles’ charges. As can be observed in Figure 1C, the NPs were stable in solution; indeed, they did not form aggregates but were evenly dispersed in liquid, showing a negative zeta potential of −21.4 mV.

**Figure 2:**
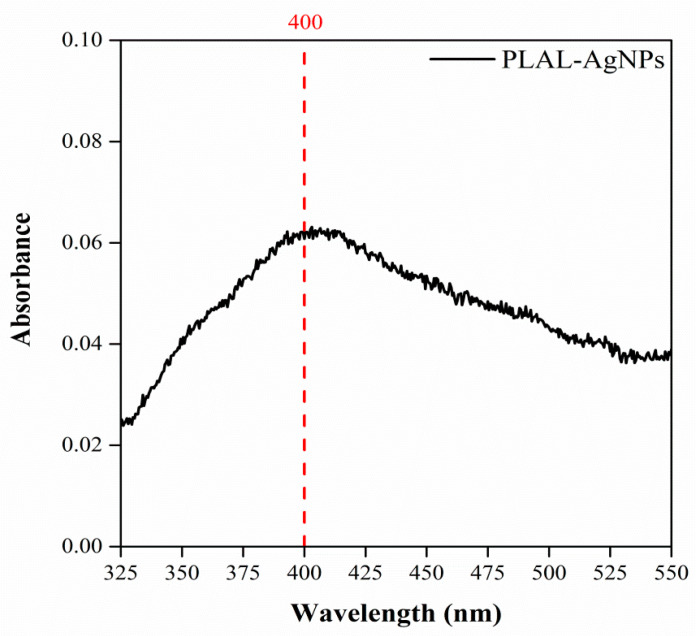
UV-vis absorption spectrum of PLAL-AgNPs in phosphate-buffered solution at 25 °C. PLAL-AgNPs showed a surface plasmon resonance band at approximately 400 nm.

A UV-Vis spectrophotometer was used to investigate the optical properties of the PLAL-AgNPs, as their behavior varies based on particle size, showing a distinct UV–visible extinction band. This band arises when incident photons of a specific frequency excite the conduction electrons collectively, a phenomenon known as localized surface plasmon resonance (LSPR) [32]. The peak wavelength of the LSPR spectrum can be directly influenced by the shape, size, morphology, and composition of silver nanoparticles. Therefore, the formation of AgNPs was monitored by measuring the surface plasmon resonance (SPR) across a wavelength range of 300–800 nm. Previous studies reported that AgNPs exhibit absorption ranging from 380 to 470 nm in UV-VIS spectra [33]. Figure 2 illustrates the absorption band of the PLAL-AgNPs, and the findings revealed a distinct surface plasmon resonance (SPR) peak at approximately 400 nm, indicating that the laser ablation method yielded purer and more homogeneous silver nanoparticles than those obtained through traditional chemical synthesis, which often requires multiple purification steps.

**Figure 3:**
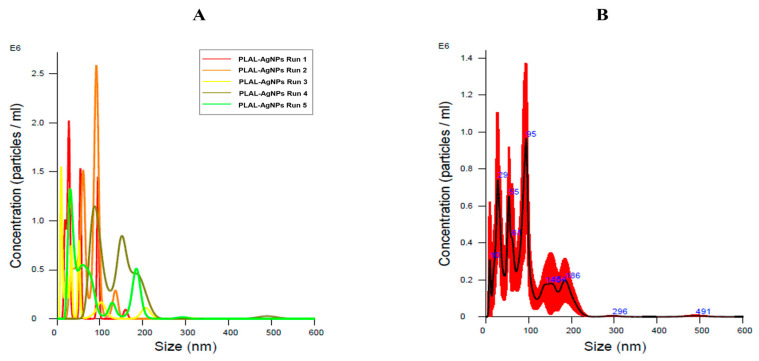
Nanoparticle tracking analysis (NTA) of PLAL-AgNPs in a 1 mL sample. (**A**) Hydrodynamic diameter size per concentration, obtained using the finite track length adjustment (FTLA) algorithm, with quintuplicate measurements (one to five runs) of the PLAL-AgNPs sample. (**B**) Average of the five measurements. All measurements were acquired using a dilution of 1:4 in PBS 1×. All the data indicate good sample reproducibility. Black line: medium value of the five measurements; red line: standard deviation.

To investigate the concentration of PLAL-AgNPs in 1 mL of sample, NTA analysis was executed (Figure 3). In addition to providing a concentration, the tool also confirmed the TEM data on the size of the PLAL-AgNPs. Indeed, the NPs consisted of 2.3 × 10^8^ particles/mL, with an average diameter of ~82.5 ± 16.9 nm and a mode value of ~49.5 ± 16.9 nm. Knowing the number of PLAL-AgNPs present in 1 mL of sample, with the help of a mathematical proportion, we obtained the number of PLAL-AgNPs present in the microliters of sample used to perform the cytotoxicity, antiviral, and antibacterial activity assays, as shown in Supplementary Table S1.

### 3.2. PLAL-AgNP Cytotoxicity Evaluation

**Figure 4:**
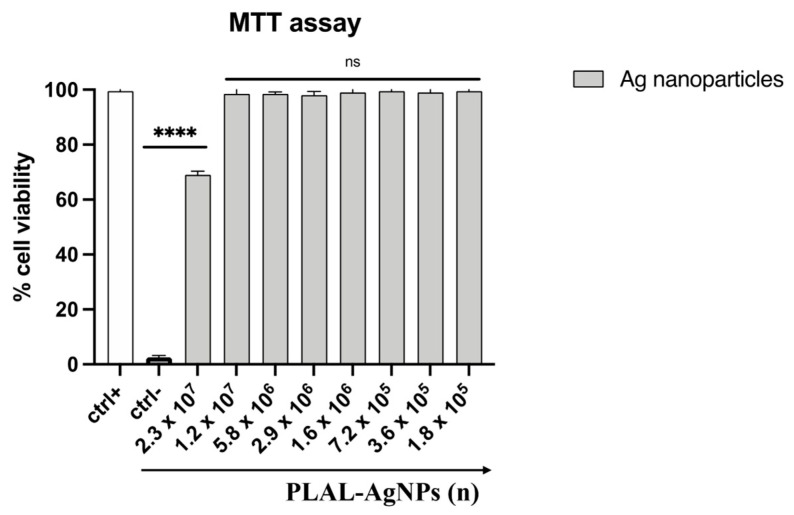
Cytotoxicity of PLAL-AgNPs on Vero cells. The percentage of cell viability was evaluated by MTT assay. The cell monolayers were treated with NPs (from 2.3 × 10^7^ to 1.8 × 10^5^) for 24 h. Untreated cells correspond to the ctrl+ of the experiment, whereas DMSO (100%) was used as a negative control (ctrl-). Data represent means ± standard deviations (SDs) of three independent experiments. ****: *p*-value < 0.0001; ns: non-significant.

The cytotoxicity of PLAL-AgNPs was evaluated by MTT assay to exclude that any antimicrobial effect could be related to toxicity on cells. In brief, Vero cells were seeded in a 96-well plate, and the next day, PLAL-AgNPs in a range of concentrations from 2.3 × 10^7^ to 1.8 × 10^5^ were added to the cell monolayers. After 24 h, the NPs were gently aspirated and an MTT solution was added for 3 h. Finally, DMSO (100%) was used to solubilize formazan crystals, and the absorbance was read at 570 nm. As reported in Figure 4, no amount of PLAL-AgNPs caused a toxic effect on the cell monolayers. 

In detail, 70% viability was observed for the highest amount of nanoparticles tested (2.3 × 10^7^). Therefore, these data indicated that this quantity of NPs could be used in subsequent experiments to investigate their antimicrobial potential.

### 3.3. Evaluation of Antiviral Activity

#### 3.3.1. HSV-1 

**Figure 5:**
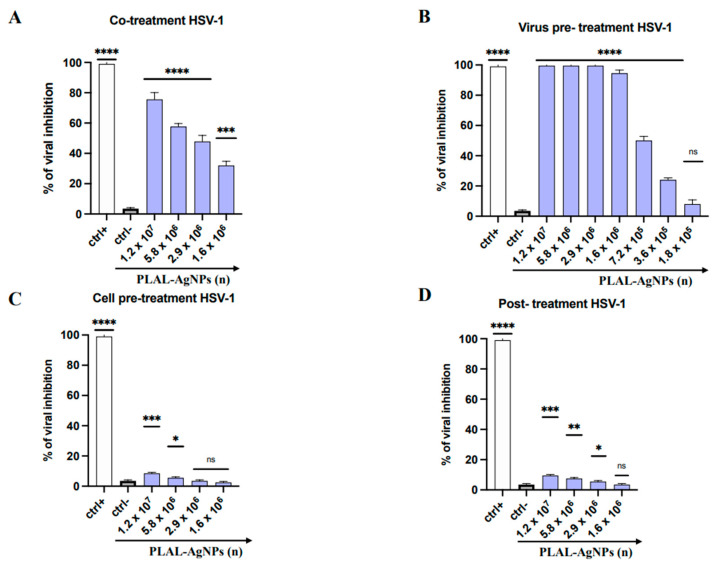
Antiviral activity of PLAL-AgNPs against HSV-1 infection: (**A**) co-treatment; (**B**) virus pre-treatment; (**C**) cell pre-treatment; (**D**) post-treatment. Different compounds were used as a positive control (ctr+) for each treatment: melittin ((**A**,**B**), 5 μM for both assays), dextran sulfate ((**C**), 1 μM), and aciclovir ((**D**), 5 μM) [34]. At the same time, the infected cells were used as a negative control (ctrl-). Data represent the means ± standard deviations (SDs) of three independent experiments. **** < 0.0001, *** = 0.0002, ** = 0.0015, * = 0.0191; ns: non-significant.

The antiviral activity of PLAL-AgNPs was investigated against HSV-1 by plaque reduction assays. In detail, four antiviral treatments were carried out to evaluate the antiviral effect of NPs (Figure 5).

In the co-treatment assay, the virus (MOI of 0.01) and AgNPs (ranging from 1.2 × 10^7^ to 1.6 × 10^6^) were added simultaneously on the cell monolayer and incubated for 1 h (the adsorption time of the virus) at 37 °C in the presence of 5% CO_2_. Subsequently, the mixture was gently removed, the cell monolayer was washed with citrate buffer (pH 3), and CMC was added. After 48 h post-infection, the cell monolayer was washed, fixed, and stained, as described in Section 2.6. The plaques were counted and compared to infected cells (ctrl-). The highest amount of PLAL-AgNPs exhibited a remarkable inhibitory effect with 70% and 60% inhibition at 1.2 × 10^7^ and 5.8 × 10^6^, respectively. In addition, the half-maximal inhibitory concentration (IC_50_) was observed at 2.9 × 10^6^ (Figure 5A). 

**Figure 6:**
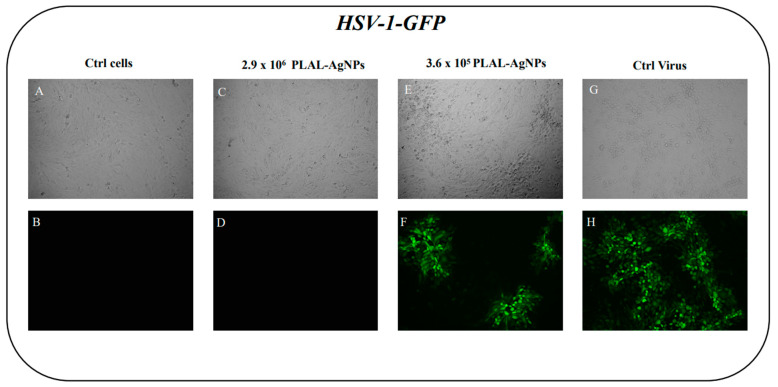
Antiviral activity of PLAL-AgNPs against HSV-1-GFP. Fluorescent and RGB images are shown. (**A**,**B**) Untreated and uninfected cells. (**C**,**D**) No plaques or GFP signals were present when cells were treated with 2.9 × 10^6^ PLAL-AgNPs. (**E**,**F**) Plaques in cells treated with 3.6 × 10^5^ AgNPs. (**G**,**H**) Control infected cells.

To investigate the mechanism of action of PLAL-AgNPs against HSV-1, other assays were conducted. In the virus pre-treatment assay, in which the virus (MOI of 0.1) and PLAL-AgNPs were first incubated together for 1 h at 37 °C and then diluted on the cell monolayer, we observed a very strong antiviral activity of PLAL-AgNPs which completely blocked HSV-1 infection until 1.6 × 10^6^ (Figure 5B). On the other hand, a decrease in the number of PLAL-AgNPs caused lower antiviral activity (20% at 3.6 × 10^5^). Finally, the cell pre-treatment (Figure 5C) and post-treatment (Figure 6D) were conducted changing the time of addition of AgNPs. In the cell pre-treatment, PLAL-AgNPs were incubated with the cells before and after the cells were infected; on the contrary, in the post-treatment, PLAL-AgNPs were added to infected cells. As shown in Figure 6C,D, no inhibition of viral replication was detected. These data confirmed that PLAL-AgNPs did not interfere with cellular mechanisms (Figure 5C) or with HSV-1 replication (Figure 5D), indicating that the potential target of PLAL-AgNPs was the viral surface. Probably, the NPs could induce direct damage to the viral particles in a very early phase of infection.

In addition, a fluorescence microscope was employed to further confirm the antiviral activity of PLAL-AgNPs against HSV-1. A fluorescent virus was used, namely, an HSV-1-GFP containing the green fluorescent protein (GFP) encoding for tegument VP22 protein, which caused infected cells to fluoresce green. In detail, a virus pre-treatment assay was conducted using an active dose of PLAL-AgNPs (2.9 × 10^6^) and a number of non-active NPs (3.6 × 10^5^). As reported in Figure 6, no fluorescent signal was observed at 2.9 × 10^6^ PLAL-AgNPs, confirming the inhibitory activity of the NPs. On the contrary, the GFP signal was evident at 3.6 × 10^5^. These results confirmed the data obtained by plaque reduction assays. 

**Figure 7:**
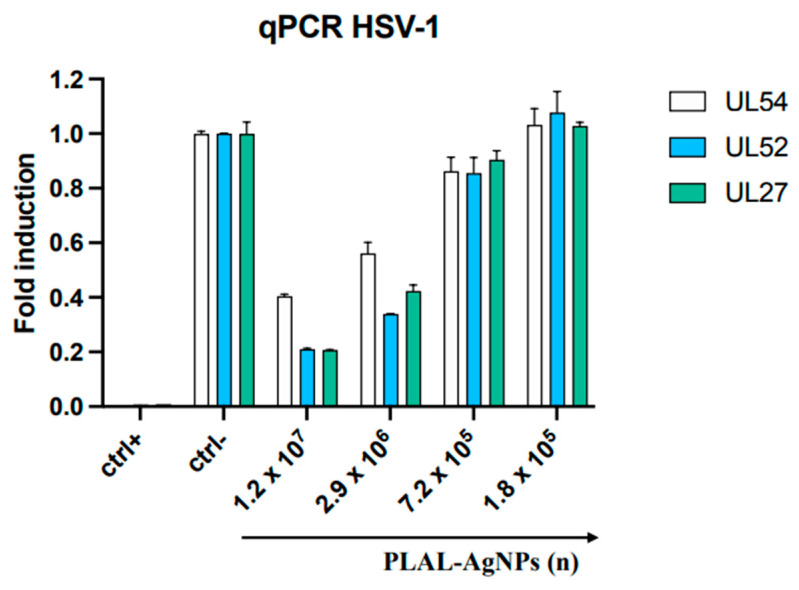
Molecular assay. Real-time PCR was conducted to evaluate the effect of AgNPs on viral gene expression. Virus pre-treatment assay was performed, RNA was extracted after 30 h, then it was retrotranscribed into cDNA and amplified. The expression of UL54, UL52, and UL27 was analyzed. Infected cells represent the ctrl-; uninfected cells represent the ctrl+.

However, the antiviral activity of PLAL-AgNPs was also evaluated by quantifying the expression levels of genes involved in HSV-1 infection. In detail, three genes were analyzed: the IE gene UL54 coding for the protein ICP 27, the E gene UL52 coding for DNA primase, and the L gene UL27 coding for structural glycoprotein B [35]. Firstly, a virus pre-treatment assay was conducted as described previously. Viral RNA was collected after 30 h of infection, converted to cDNA by retrotranscription, and amplified by real-time PCR. The results reported in Figure 7 show that amounts of PLAL-AgNPs interfered with viral replication by reducing the levels of expression of the three genes in a dose-dependent manner. On the contrary, treating cells with non-active amounts of PLAL-AgNPs did not interfere with the levels of gene expression, so they reached the same levels as those of the virus control (ctrl-).

#### 3.3.2. PV-1

**Figure 8:**
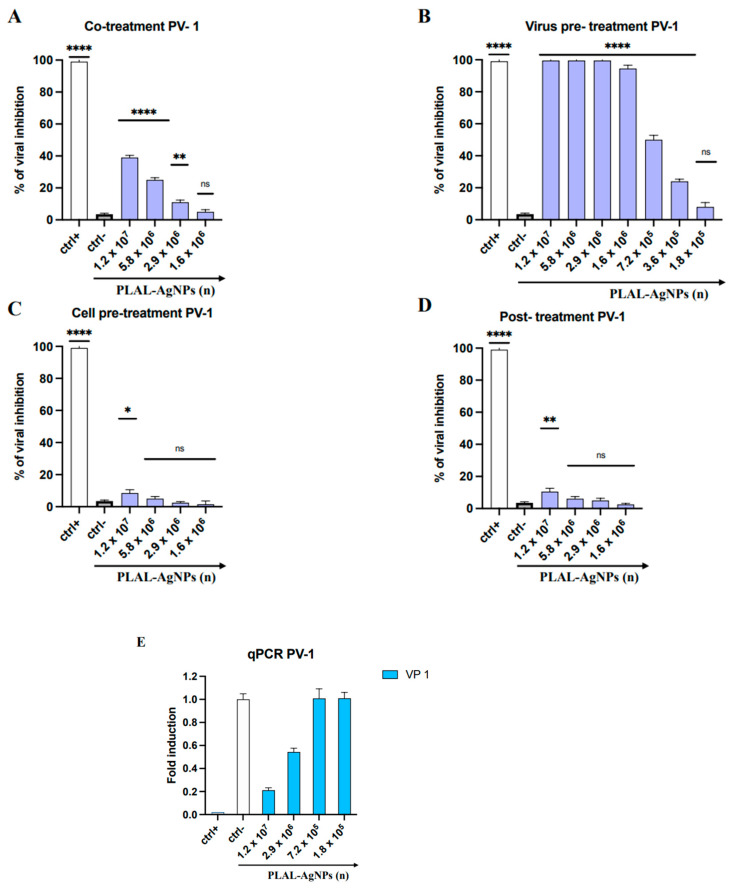
Antiviral activity of PLAL-AgNPs against PV-1 infection: (**A**) co-treatment; (**B**) virus pre-treatment; (**C**) cell pre-treatment; (**D**) post-treatment. Different compounds were used as positive controls (ctr+) for each treatment: pleconaril ((**A**,**B**), 2 μg/mL for both assays) [37], WIN51711 ((**C**) 5 μg/mL), and protein 2C ((**D**), 10 μM) [38], while infected cells were used as negative controls (ctr-). Data represent the means ± standard deviations (SDs) of three independent experiments. **** < 0.0001, ** = 0.0032, * = 0.0457; ns = non-significant. (**E**) Molecular assay. Real-time PCR was performed to evaluate the effect of AgNPs on viral gene expression. Virus pre-treatment assay was performed, RNA was extracted after 24 h, then retrotranscribed into cDNA and amplified. Gene expression of the capsid protein VP1 was evaluated. Infected cells represent the ctrl-, while uninfected cells represent the ctrl+.

The antiviral activity of PLAL-AgNPs was also investigated against PV-1, a virus different from HSV-1 since it is a naked virus with an RNA genome. Here, we observed the strongest antiviral activity in a virus pre-treatment assay by incubating PLAL-AgNPs with the virus and then treating cells with the mixture, while only a slight inhibitory effect was detected in a co-treatment assay (Figure 8A). 

In detail, 100% inhibition was observed when cells were treated with a number of PLAL-AgNPs from 1.2 × 10^7^ to 2.9 × 10^6^ (Figure 8B). The IC_50_ was at 7.2 × 10^5^. A lower amount of PLAL-AgNPs did not show inhibitory activity. No antiviral activity was observed in the cell pre-treatment and post-treatment assays (Figure 8C,D), indicating that PLAL-AgNPs did not interfere with the cellular surface and viral replication. 

As reported in the literature, Huy et al. indicated that the difference in size between AgNPs and viral particles is very important in order to determine the antiviral effect: the AgNPs, synthetized by an electrochemical method, had a size of 7.1 nm and reduced viral infection at the lowest tested concentration of 3.13 ppm [36]. Perhaps this depended on the size of the NPs; most likely, the smaller ones interacted with the viral particles of PV-1 that were about 25–30 nm in diameter. This difference provided great mobility to the AgNPs, and thus they easily interacted with poliovirus particles. 

To further confirm the antiviral activity of PLAL-AgNPs against PV-1, gene expression levels of the capsid protein VP1 were evaluated using real-time PCR. As reported in Figure 8E, PLAL-AgNPs inhibited the expression of the protein in a dose-dependent manner. 

### 3.4. Antibacterial Activity

#### 3.4.1. MIC and Time–Killing Assay

**Figure 9:**
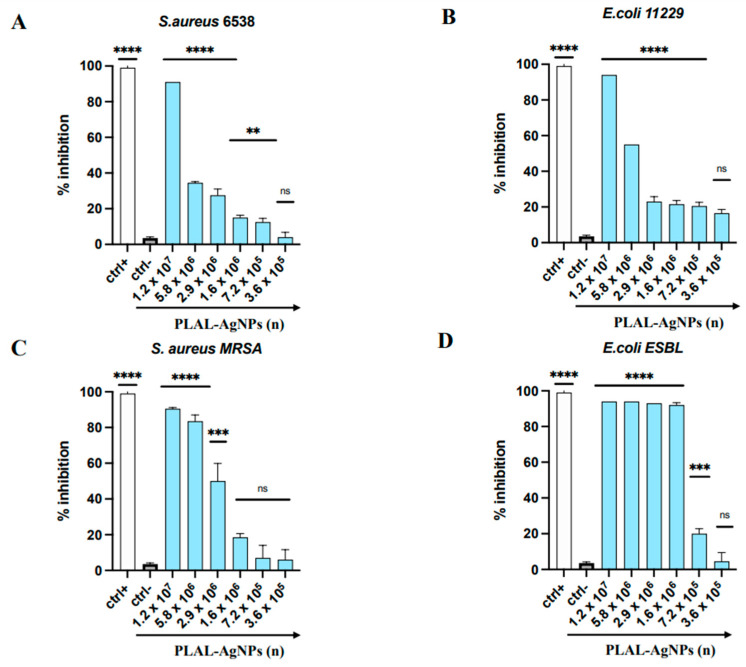
Antibacterial activity of PLAL-AgNPs against (**A**) *S. aureus* 6538, (**B**) *E. coli* 11229, (**C**) *S. aureus* MDR, and *(***D**) *E. coli* ESBL. Statistical significance was referred to the negative control and determined by analysis of variance (ANOVA). Dunnett’s test was used for multiple comparisons with controls. ****: *p-*value < 0.0001, ***: *p-*value = 0.0004, **: *p-*value = 0.0055; ns: *p-*value = non-significant.

The antibacterial potential of PLAL-AgNPs was evaluated against *S. aureus* 6538 and *E. coli* 11229 (as representatives of Gram-positive and Gram-negative bacteria, respectively) and against two clinical strains, i.e., *S. aureus* MDR and *E. coli* ESBL, by microdilution assay (Figure 9). 

In detail, 1.2 × 10^7^ PLAL-AgNPs were able to inhibit the growth of *S. aureus* (Figure 9A) and *E. coli* (Figure 9B); 30% (*S. aureus*) and 50% (*E. coli*) inhibition was observed by using 5.8 × 10^6^ PLAL-AgNPs. The lower amount of AgNPs inhibited growth with an inhibition rate of less than 20%.

On the contrary, we found a strong antibacterial activity against the two clinical isolates. The PLAL-AgNPs showed an MIC at 5.8 × 10^6^ against the clinical isolate of *S. aureus* (Figure 9C). Also, in this case, using a lower amount of PLAL-AgNPs, the bacterial inhibition rate decreased by 20%. Meanwhile, a strong antibacterial activity was detected against the clinical strain of *E. coli* (Figure 9D), with an MIC at 1.6 × 10^6^.

**Figure 10:**
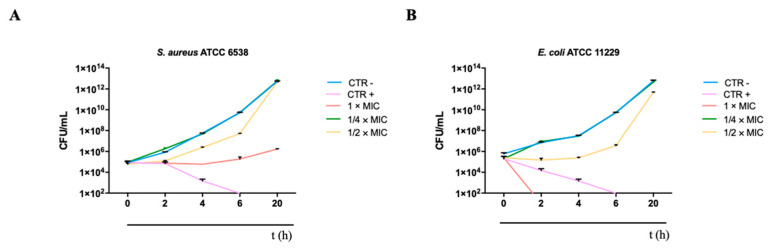
Killing kinetics of AgNPs against (**A**) *S. aureus* 6538 and (**B**) *E. coli* 11229.

In addition, these results were confirmed by monitoring bacterial growth over time. The time–killing assay was conducted against *S. aureus* and *E. coli* (ATCC strains) and showed a different trend (Figure 10). 

The curve for untreated bacteria increased exponentially over time. Treatment with 5.8 × 10^6^ (1/2 MIC) and 2.9 × 10^6^ (1/4 MIC) PLAL-AgNPs did not induce significant changes in bacterial growth, showing viable cell counts comparable to the negative control. On the other hand, bacterial cells of *E. coli* treated with 5.8 × 10^6^ (1/2 MIC) and 2.9 × 10^6^ (1/4 MIC) AgNPs were comparable to the untreated cells (ctrl-), while, by using 1 × 10^7^ PLAL-AgNPs (MIC), we observed a complete eradication of the bacterial load after about 2 h, indicating bactericidal action.

#### 3.4.2. SEM Analysis

The antibacterial activity of PLAL-AgNPs against Gram-positive and Gram-negative bacteria was further investigated by SEM. Bacteria were treated with 1.2 × 10^7^ (MIC) and 5.8 × 10^6^ (½MIC) PLAL-AgNPs. After being fixed in glutaraldehyde and dehydrated with ethanol solution at increasing concentrations (25, 50, 70, 95, and 100% *v*/*v*), PLAL-AgNPs were observed via SEM. 

**Figure 11:**
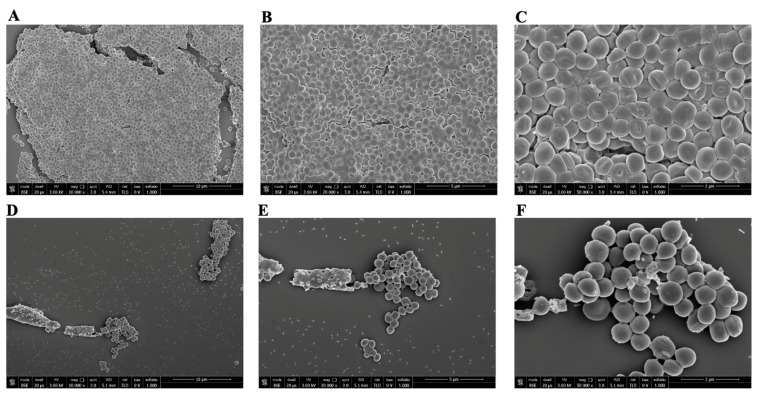

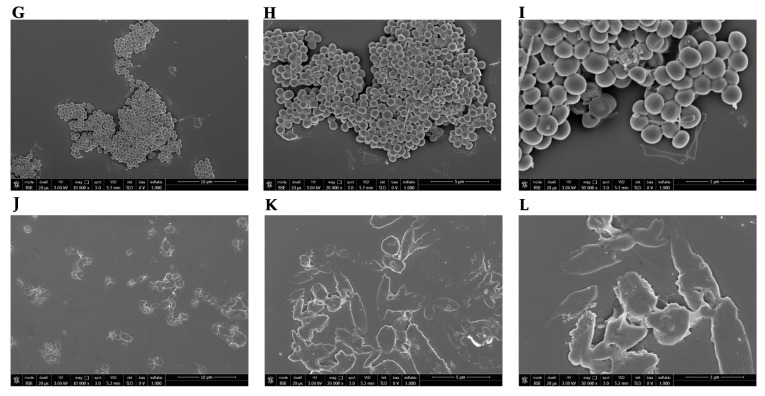
SEM visualization of PLAL-AgNPs and *S. aureus*. (**A**–**C**) Untreated *S. aureus* 6538 cells (ctrl-) at a magnification of 10,000, 20,000, and 50,000×, respectively. (**D**–**F**) *S. aureus* treated with 1.2 × 10^7^ PLAL-AgNPs (MIC) at a magnification of 10,000, 20,000, and 50,000×, respectively. (**G**–**I**) *S. aureus* treated with 5.8 × 10^6^ PLAL-AgNPs (½ MIC) at a magnification of 10,000, 20,000, and 50,000×, respectively. (**J**–**L**) *S. aureus* treated with 2 μg/mL of vancomycin (ctrl+) at a magnification of 10,000, 20,000, and 50,000×, respectively.

**Figure 12:**
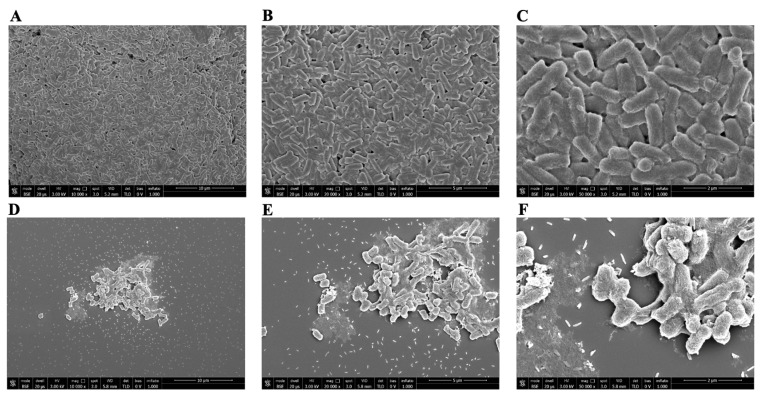

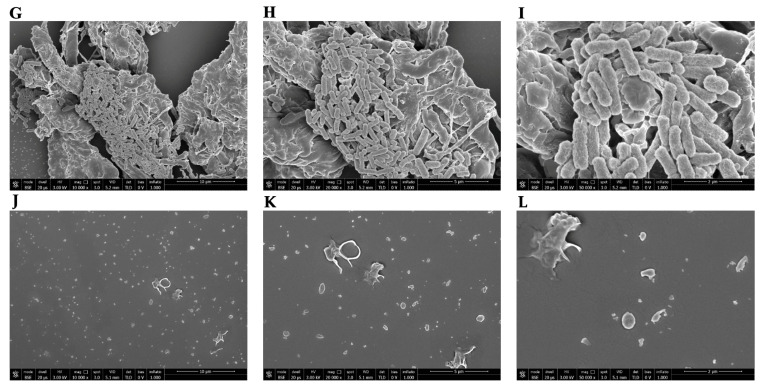
SEM visualization of PLAL-AgNPs and *E. coli*. (**A**–**C**) Untreated *E. coli* 11229 cells (ctrl-) at a magnification of 10,000, 20,000, and 50,000×, respectively. (**D**–**F**) *E. coli* treated with 1.2 × 10^7^ PLAL-AgNPs (MIC) at a magnification of 10,000, 20,000, and 50,000×, respectively. (**G**–**I**) *E. coli* treated with 5.8 × 10^6^ PLAL-AgNPs (½ MIC) at a magnification of 10,000, 20,000, and 50,000×, respectively. (**J**–**L**) *E. coli* cells treated with 8 μg/mL of amikacin (ctrl+) at a magnification of 10,000, 20,000, and 50,000×, respectively.

In Figure 11 and Figure 12, we observed structural damage when *S. aureus* and *E. coli* were treated with PLAL-AgNPs at MIC concentrations. The bactericidal action of AgNPs against *E. coli* cells was confirmed, since the morphological damage and presence of cellular debris that we observed at the MIC concentration were more evident in *E. coli* than in *S. aureus*, thus PLAL-AgNPs showing a bacteriostatic action.

### 3.5. Antibiofilm Activity

**Figure 13:**
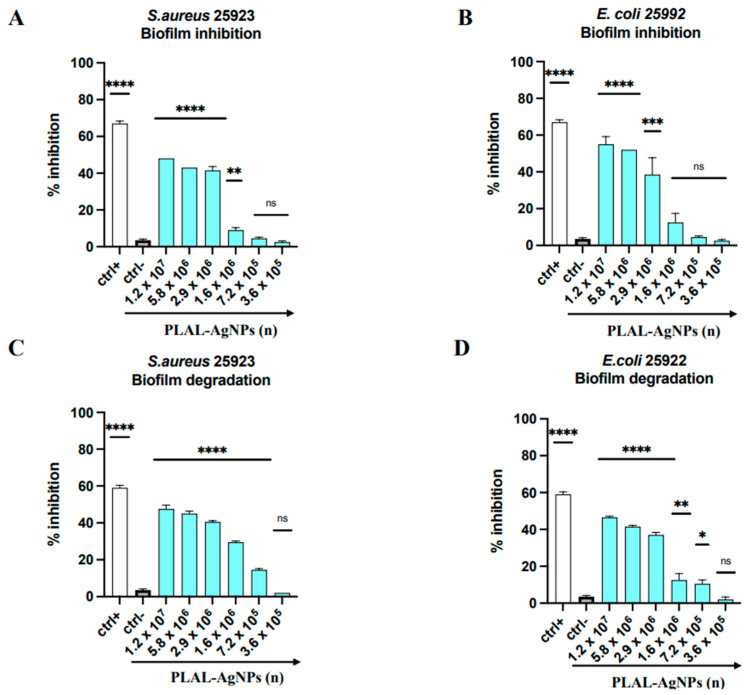
Antibiofilm activity of PLAL-AgNPs. Biofilm inhibition and degradation against *S. aureus* (**A**,**C**). Biofilm inhibition and degradation against *E. coli* (**B**,**D**). Data represent the means ± standard deviations (SDs) of three independent experiments. ****: *p-*value < 0.0001, ***: *p-*value = 0.0001, **: *p-*value = 0.0058, *: *p-*value = 0.0159; ns: non-significant.

The present investigation aimed to evaluate the in vitro antibiofilm efficacy of PLAL-AgNPs against biofilm-forming bacteria, particularly *E. coli* and *S. aureus*. Each mature and forming biofilm was exposed to increasing concentrations of NPs ranging from 1.2 × 10^7^ to 3.6 × 10^5^. Biofilm stands as a critical virulence determinant owing to its role in impeding antibiotic absorption, harboring dormant bacteria unaffected by conventional antibiotics, and facilitating genetic exchange of virulence determinants. Consequently, the development of a novel strategy capable of disrupting this structure signifies a paramount objective [22]. The results revealed that the biosynthesized PLAL-AgNPs effectively prevent biofilm formation by bacterial species and affect mature biofilm, showing a notable contrast with the negative control employed in the experiment. After subjecting *E. coli* and *S. aureus* to the highest dosage of PLAL-AgNPs (1.2 × 10^7^) for a duration of 24 h, a reduction in biofilm formation of 45% and 53%, respectively, was observed in comparison to untreated biofilm samples (Figure 13A,B and Supplementary Figure S1). 

On the other hand, a disruption rate of 46 and 48% was observed when the mature biofilms of Gram-positive and Gram-negative strains were exposed to 1.2 × 10^7^ PLAL-AgNPs, respectively (Figure 13C,D and Supplementary Figure S1). Investigations into the antibiofilm properties of AgNPs remain relatively scarce. Bacterial biofilm formation results from bacterial adhesion to both biotic and abiotic surfaces coupled with the synthesis and secretion of exopolysaccharides (EPSs) by bacterial cells [39]. Bacteria adhere to surfaces and, triggered by environmental cues, prompt the synthesis of exopolysaccharides (EPSs) [40]. Consequently, the inhibition or prevention of these processes would consequently restrict biofilm formation. Siddique et al. evaluated the efficacy of inhibition of Klebsiella pneumoniae biofilm formation in response to treatment with AgNPs synthesized using sodium borohydride (NaBH4) and stabilized with polyvinylpyrrolidone (PVP). At a concentration of 100 µg/mL of AgNPs, the average percentage of biofilm inhibition was 75%. The authors attributed this antibiofilm activity to the decrease in EPSs. In fact, treatment with AgNPs led to an average reduction of 35.8% in the amount of EPSs [41]. Likewise, Tabassum et al. demonstrated substantial inhibition of biofilm formation in *Pseudomonas aeruginosa*, *Klebsiella pneumoniae*, *Candida albicans*, *E. coli*, *Listeria monocytogenes*, *S. aureus*, and *Streptococcus mutans* when exposed to sub-MIC levels of PVD-AgNPs. The observed reductions in biofilm formation, ranging from 40% to 90%, resulted primarily from the downregulation of genes associated with exopolysaccharide biosynthesis, particularly algA and algU. Moreover, Montazeri and collaborators showed a notable decrease in the expression levels of the genes associated with the biofilm adhesion phases icaA and icaD (*S. aureus*) by an average of 63.5% when exposed to sub-inhibitory concentrations of AgNPs [42]. Also, Swolana et al. demonstrated the involvement of AgNPs in impacting the expression of the icaADBC operon and the icaR gene [43]. The formation and structural integrity of biofilm is sustained through a combination of quorum sensing (QS) mechanisms and intermolecular interactions among its diverse components. In support of this, Al-Momani and colleagues provided evidence by examining the impact of AgNPs on the expression of QS-regulated genes, such as lasI, lasR, rhlI, rhlR, pqsR, and pqsA (*Pseudomonas aeruginosa*). They observed a significant reduction in the expression of these genes at an AgNP concentration of 7.5 µg/mL, which disrupted the QS system. However, it is crucial to recognize that biofilm development is a dynamic process in which QS activity not only influences the initial formation phase but more importantly plays a critical role in maintaining biofilm integrity through subsequent cell proliferation and post-expansion inoculation. Consequently, interference with the QS system led to the destabilization of the biofilm structure [44]. Additionally, Kraśniewska et al. documented that the incorporation of silver nanoparticles (AgNPs) into biofilm matrices could attenuate the intermolecular interactions among their constituents, consequently reducing the surface tension of the films [45]. The results underlined the in vitro antibiofilm efficacy of PLAL-AgNPs and their potential in counteracting E. coli and S. aureus infections. Subsequent investigations will prioritize unraveling the precise mechanisms through which these nanoparticles disrupt the dangerous microbial ecosystem.

## 4. Conclusions

The present study reported the antimicrobial potential of PLAL-AgNPs synthesized by an innovative physical top-down approach called PLAL. The PLAL-AgNPs were characterized by TEM, zeta potential, X-ray fluorescence, and NTA analyses. They showed no toxic effects on the cell model used. Antiviral activity was evaluated against different viruses, including the enveloped HSV-1 and the naked PV-1. Our data demonstrated that the PLAL-AgNPs interfered directly with viral particles acting in the early stage of viral infection.

The antibacterial activity was evaluated against bacterial pathogens for humans and also against clinical isolates to understand if the nanoparticles could limit antibiotic resistance phenomena. The PLAL-AgNPs showed a bacteriostatic effect against *S. aureus* and a bactericidal effect against *E. coli* in the early stages of exposure. The multiplicity of actions of these PLAL-AgNPs suggested that they could represent promising broad-spectrum antimicrobial agents. However, other studies will be necessary to deepen understanding of their mechanism of action.

## Data Availability

Data are contained within the article.

## Supplementary Materials

The following supporting information can be downloaded at: https://www.mdpi.com/article/10.3390/microorganisms12040820/s1, Figure S1: Inhibition and degradation assay of *S. aureus* ATCC 25923 and *E. coli* ATCC 25992 biofilms exposed to AgNPs; Table S1: Number of PLAL-AgNPs in μL of sample.
