# Supplementary material for: Ligand-Free Silver Nanoparticles: An Innovative Strategy against Viruses and Bacteria
Source: Microorganisms. 2024 Apr 18;12(4):820. doi: 10.3390/microorganisms12040820 (PMC11052337; doi:10.3390/microorganisms12040820)
Supplement: Supplementary file 1 [file microorganisms-12-00820-s001.zip › microorganisms-2936626-supplementary.pdf]

Supplementary Table S1. Number of PLAL-AgNPs in µL of sample.

AgNPs microliters (µL)	AgNPs number
100	2.3×10^7
50	1.2×10^7
25	5.8×10^6
12.5	2.9×10^6
6.3	1.6×10^6
3.1	7.2×10^5
1.6	3.6×10^5
0.8	1.8×10^5

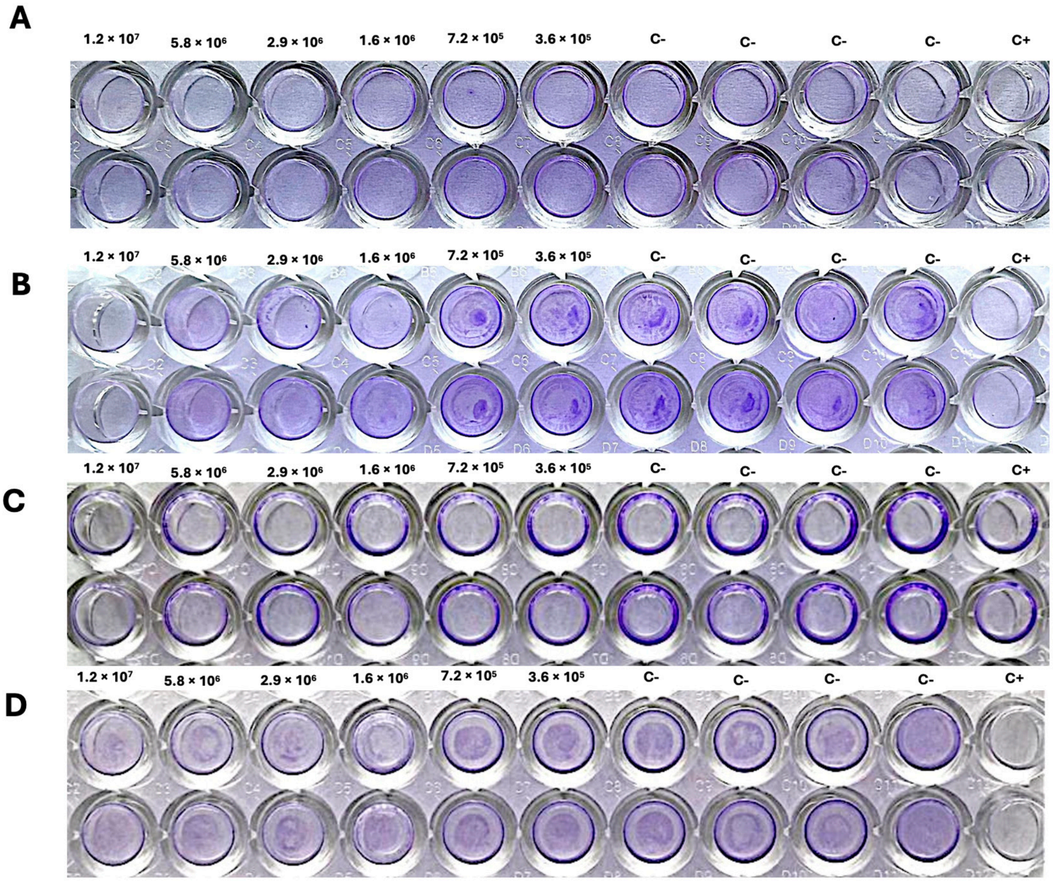

Supplementary Figure S1. Inhibition (A and C) and degradation (B and D) assay of *S. aureus* ATCC 25923 and *E. coli* ATCC 25992 biofilms exposed to AgNPs.
